# Variance estimation in reference based sensitivity analysis for longitudinal trials with protocol deviation

**DOI:** 10.1186/1745-6215-16-S2-P127

**Published:** 2015-11-16

**Authors:** Suzie Cro, Michael Kenward, James Carpenter

**Affiliations:** 1 MRC Clinical Trials Unit at UCL, London, UK; 2 London School of Hygiene and Tropical Medicine, London, UK

## 

The statistical analysis of longitudinal randomised clinical trials is frequently complicated by the occurrence of protocol deviations, which result in incomplete data sets for analysis. Analysis and inference then rest on inherently untestable assumptions about the distribution of the unobserved data. It is therefore important to perform sensitivity analysis to explore the robustness of conclusions from the primary analysis to a range of contextually plausible assumptions about the missing data.

Carpenter, Roger and Kenward (2013) propose a novel pattern-mixture approach for contextually relevant sensitivity analysis of a longitudinal trial. Their proposal uses multiple imputation to impute post-deviation data by reference to patients in other trial arms. This flexible approach avoids the need for users to specify explicitly the distribution of the post-deviation data. The primary analysis model is retained in the sensitivity analysis allowing direct assessment of the impact of alternative sampling behaviour on the primary analysis conclusions.

Reference based sensitivity analysis uses Rubin's multiple imputation variance rules. However it is unclear precisely what Rubin's variance formula is estimating when there is a mismatch between the imputation and analysis models.

We present theoretical and simulation results that show, in this context, Rubin's rules approximately preserve the fraction of missing information across a range of sensitivity analysis assumptions. This provides a solid justification for their practical use. We also present a new Stata command “mimix” that conducts reference based multiple imputation for sensitivity analysis of clinical trials with protocol deviation.

